# Sensation of presence and cybersickness in applications of virtual reality for advanced rehabilitation

**DOI:** 10.1186/1743-0003-4-34

**Published:** 2007-09-25

**Authors:** Tohru Kiryu, Richard HY So

**Affiliations:** 1 Graduate School of Science and Technology, Niigata University, Niigata, Japan; 2 Department of Industrial Engineering and Logistics Management, Hon Kong University of Science and Technology, Hong Kong SAR, PR China

## Abstract

Around three years ago, in the special issue on augmented and virtual reality in rehabilitation, the topics of simulator sickness was briefly discussed in relation to vestibular rehabilitation. Simulator sickness with virtual reality applications have also been referred to as visually induced motion sickness or cybersickness. Recently, study on cybersickness has been reported in entertainment, training, game, and medical environment in several journals. Virtual stimuli can enlarge sensation of presence, but they sometimes also evoke unpleasant sensation. In order to safely apply augmented and virtual reality for long-term rehabilitation treatment, sensation of presence and cybersickness should be appropriately controlled. This issue presents the results of five studies conducted to evaluate visually-induced effects and speculate influences of virtual rehabilitation. In particular, the influence of visual and vestibular stimuli on cardiovascular responses are reported in terms of academic contribution.

## Localization of Advanced Rehabilitation

**Figure 1:**
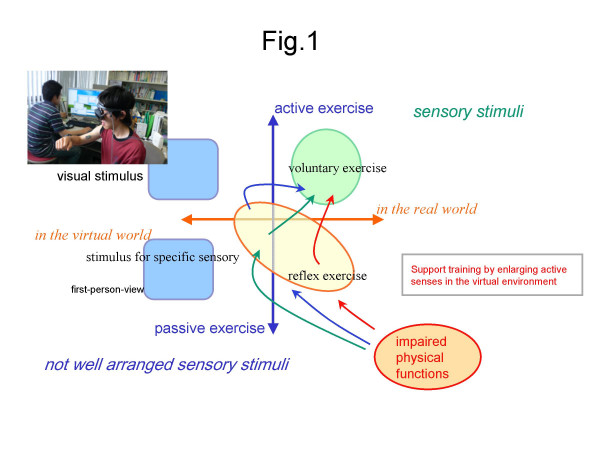
Recently proposed approaches in advanced rehabilitation according to the type of motion controls (active or passive) and the space of interactions (real or virtual).

Sensory and physical assistive devices have long been developed to support impaired functions in patients. Even a powered-suit has recently been developed to strengthen muscle force [1]. Besides, current virtual reality (VR) technology expands not only sensory effects but also physical activities, and the potential effects are expected in rehabilitation engineering [2]. The expecting challenge has been on how to create or promote regular exercises for a variety of individual physical conditions. Figure 1 illustrates recently proposed approaches in advanced rehabilitation according to the type of motor controls (active or passive) and the space of interactions (real or virtual). As shown in Figure 1, active or voluntary physical exercise in the real world increase one's fitness or wellness. However, it needs continuous motivation to keep a habit of regular physical exercise, because people hate sweat and boring repetitive training or exercise. Thus applications to facilitate passive exercises in the real world emerge in the business of health promotion. Mechanically induced motion or electrical stimulations on muscles produce passive exercise. During active exercise, muscles contractions are activated by neural impulses from the brain via the spinal cord to produce voluntary exercise. Reflex, on the other hand, is a reaction to incoming stimuli. Since reflex accompanies with muscle contractions, passive muscle contractions induced by repetitive stimuli have been used to produce passive exercise. Using VR technology, applications can be developed to allow users to experience active or passive exercises in the virtual world without little limitation. Very often, stimuli in VR applications will exceed the normal boundary experienced by users in their daily lives.

Enhancing a specific sensory stimulus, however, has been reported to evoke some unpleasant sensation due to the conflict among sensory stimuli (sensory conflict theory) [3]. This type of problems in VR applications has been referred to as cybersickness – a type of simulator sickness. In particular, multi-sensory stimuli that are inappropriate to each other or slightly different from those experienced in the real world could evoke symptoms of cybersickness, even though such stimuli would excite the users and increase their sensed feeling of reality. Thus, for expanding application of VR in rehabilitation engineering, concerns of cybersicknes should be addressed. Referring to neuroscientific models [4-6], the influences of vestibular-autonomic responses and ocular-autonomic responses on motion sickness has been suggested. Thus, the analysis as illustrated in Fig. 1 calls for studies to clarify the differences in the influences on autonomic nervous regulation during different types of exercises (real active exercise, real passive exercise, and virtual exercise).

## Background on the Behavior of Biosignals

**Figure 2:**
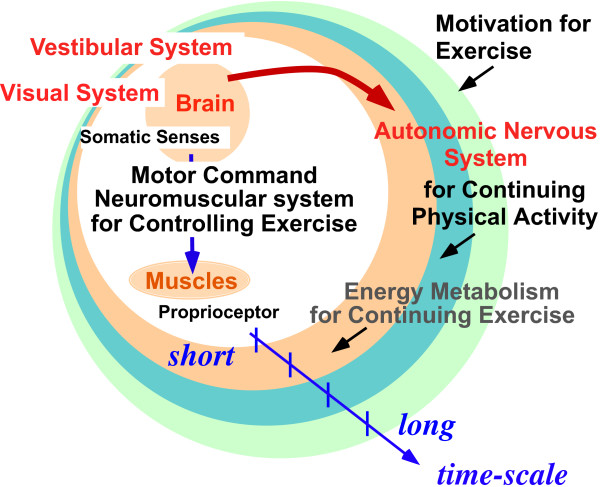
Several time-scales in biosignals during exercise [11].

The autonomic nervous regulation would be evaluated during a recovery phase because it regulates cardiovascular functions after extensive exercise or stress. That is, there is a time delay between the incoming stimuli for sensory systems and the corresponding autonomic regulation. Moreover, there is a large difference in time-scale between sensory activity and autonomic nervous activity (ANA) (Fig. 2). In particular, sensory activities work within a few tens of milliseconds, whereas ANA takes several seconds. Due to such a large difference in time-scale, researchers have studied either one or the other, but not both.

**Figure 3:**
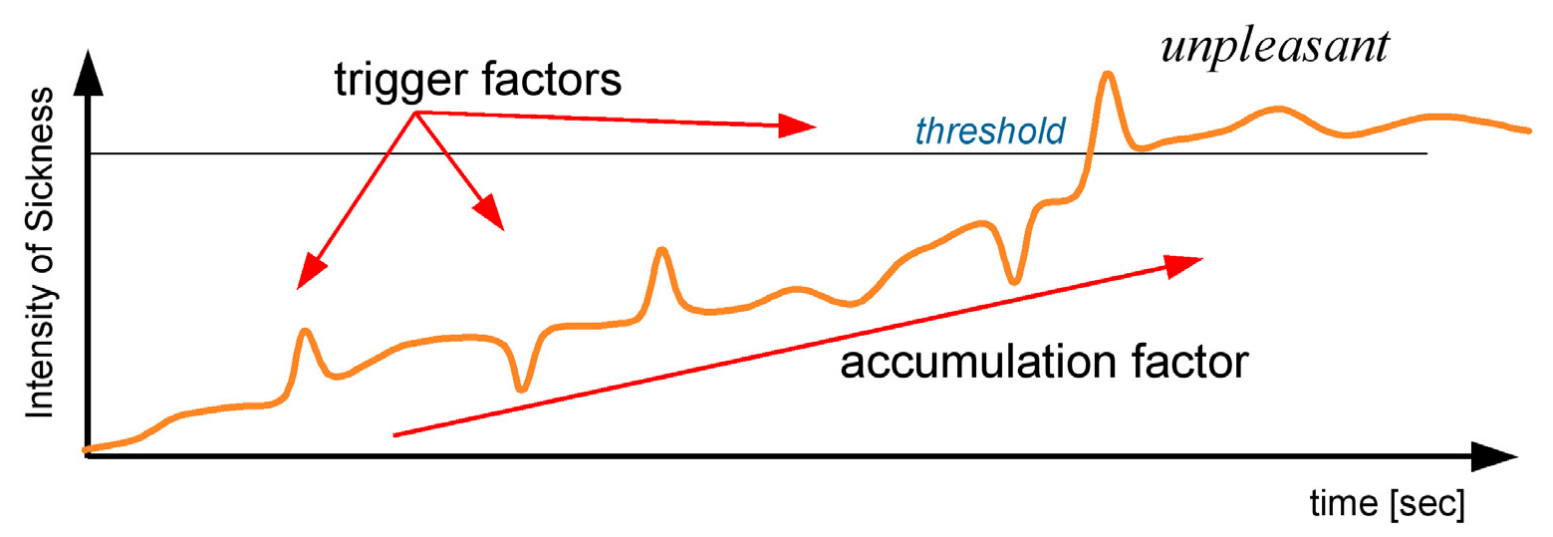
Time-varying factors model with trigger factors and accumulation factor (adapted from [7]).

In rehabilitation, repetitive task practice is a common approach to recover impaired functions. To achieve successful recovery, practice and rest periods and levels of training should be carefully controlled depending on individual differences. Figure 3 demonstrates a model in which the progress in recovery consists of an accumulation factor and trigger factors [7]. An accumulation factor has a long time scale because it relates to background ANA, while trigger factors have a short time scale because of the relatively fast sensory processing in the brain.

The trigger factors have a short time scale and are related to display devices and video images, and sensory and cognitive systems. The accumulation factor has a long time scale and is evaluated by the autonomic regulation after specific visual stimuli. Although visual stimuli might be weak, the development of symptom could occur due to the progression of time. According to our preliminary study [7], the accumulation did not simply increase with respect to time. Accumulation factor most likely links to specific trigger factors. The features and timings of specific trigger factors should be further studied. Preliminary results also suggest that different thresholds could exist between positive and negative sensations even for the same stimuli, depending on the individual capacity of autonomic regulation affected by the cardiovascular system.

Preventing unpleasant situation is a key point for sustaining sufficient effectiveness and motivation. Since the heart rate is different between virtual and real exercises, activation of muscle contraction even in virtual environment could suppress cybersickness. Further study on the difference between real and virtual exercises in terms of the time-varying factors model should reveal hints to design continuous repetitive VR rehabilitation tasks effectively.

## Measurement and Evaluation of Biosignals associated with Presence and Cybersickness

Mismatch between the visual and vestibular systems can disturb the autonomic nervous regulation and lead to symptoms of motion sickness [5]. Moreover, there is an interaction between ANA and muscular activity in terms of autonomic regulation [8]. Heart-rate variability, i.e., the fluctuation in the R-R interval derived from electrocardiograms, has been widely used to evaluate ANA during exercise [9]. In practice, the ANA-related indices have been estimated from biosignals including heart rate, blood pressure, finger pulse volume, respiration rate, skin condition, and gastric myoelectrical activity. Measured biosignals at the sensory systems were transformed into some estimated values to represent the input-output-relation in the relatively same time-scale of autonomic regulation. Sensory systems including muscles are evaluated at the input-level and the ANA are evaluated at the output-level. The amplitude and frequency indices of surface electromyograms have been used to measure muscle fatigue [10]. Since some stimuli are hard to be measured, there is the limitation of ANA-related indices estimated from measured biosignals. Then, the questionnaire was often used as a subjective index.

A certain level of quantization of sensory stimuli is now available, and large individual variations have been found. Accordingly, personalized evaluation procedures of sensory systems and autonomic regulation should be developed before an effective application of the VR technology in rehabilitation engineering can be established. Otherwise, undesirable autonomic nervous responses could accumulate to produce symptoms of cybersickness.

## Scope in this Issue

This issue presents several approaches to evaluate the effects of incoming stimulus on cardiovascular systems. Sugita et al. show how to evaluate reproducibility and adaptation of visually induced motion sickness based on the maximum cross-correlation between pulse transmission time and heart rate. They conclude that the physiological index would be effective for assessing reproducibility and adaptation of visually induced motion sickness. Regarding sensory features, Oyamada and colleagues present a pilot study on pupillary and cardiovascular reflexes induced by stereoscopic motion video movies and show that the autonomic responses, separately from the pupillary light reflex, are effective to monitor biomedical effects induced by image presentation. Then, Tanahashi et al. discuss effects of visually simulated motion stimulus on vection and postural stabilization. They speculate that there could be different thresholds in the processing of visual motion signals for postural control and vection perception. In addition, Watanabe and associates reports a preliminary study on the effect of predictive visual sign of acceleration on heart rate variability in a motion-based VR driving simulator. They demonstrate the importance of the interval between signs and events. In all of them, exercises were passive and subjects were sitting on the chair or standing while viewing motion videos. Finally, Kiryu and colleagues report a study on the differences in real active and virtual passive exercises in terms of autonomic regulation to incoming sensory and physical stimuli. Based on the results, they propose an appropriate evaluation process for handling biosignals with different time-scales.

In this issue researchers have struggled to quantitatively evaluate the visually-induced effects and influences in the fields regarding motion images, sensory systems, and autonomic nervous regulation. Valuable results and hints have been suggested for researchers who are enrolling in this field, although some findings remain preliminary. All in all, we hope that this issue will advance our understanding on the effects and influences of enhanced or augmented VR stimuli in rehabilitation applications.

## References

[B1] Kawamoto H, Lee S, Kanbe S, Sankai Y (2003). Power assist method for HAL-3 using EMG-based feedback controller. Proc of Int Conf Systems, Man and Cybernetics.

[B2] Kenyon RV, Leigh J, Keshner EA (2004). Considerations for the future development of virtual technology as a rehabilitation tool. J NeuroEng Rehab.

[B3] Reason JT, Brand JJ (1975). Motion Sickness.

[B4] Bles W, Bos JE, de Graaf B, Groen E, Wertheim AH (1998). Motion sickness: only one provocative conflict?. Brain Research Bulletin.

[B5] Yates BJ, Miller AD, Lucot JB (1998). Physiological basis and pharmacology of motion sickness: an update. Brain Research Bulletin.

[B6] Ji J, So RHY, Lor F, Cheung TFR, Howrth P, Stanney K (2005). A search for possible neural pathways leading to visually induced motion sickness. Vision.

[B7] Kiryu T, Uchiyama E, Jimbo M, Iijima A, R Shumaker (2007). Time-varying factors model with different time-scales for studying cybersickness. Virtual Reality, Human-Computer Interaction International.

[B8] Saito M, Tsukanaka A, Yanagihara D, Mano T (1993). Muscle sympathetic nerve responses to graded leg cycling. J Appl Physiol.

[B9] Anosov O, Patzak A, Kononovich Y, Persson PB (2000). High-frequency oscillations of the heart rate during ramp load reflect the human anaerobic threshold. Eur J Appl Physiol.

[B10] Merletti R, Knaflitz M, De Luca CJ (1990). Myoelectric manifestations of fatigue in voluntary and electrically elicited contractions. J Appl Physiol.

[B11] Kiryu T, Iijima A, Bando T (2005). Relationships between sensory stimuli and autonomic regulation during real and virtual exercises. Proc 27th Annu Int Conf IEEE/EMBS.

